# The development of indicators to measure the quality of care in geriatric rehabilitation

**DOI:** 10.1093/intqhc/mzad044

**Published:** 2023-09-01

**Authors:** Bram Veneberg, Lian M J Tijsen, Maarten J Wirtz, Viola Zevenhuizen, Bianca I Buijck

**Affiliations:** Department of Science and Technology, University of Twente, Drienerlolaan 5, Enschede 7522 NB, The Netherlands; Department of Public Health and Primary Care, Leiden University Medical Center, PO Box 9500, Leiden 2300 RA, The Netherlands; Oktober, Wielewaal 10, Bladel 5531 LJ, The Netherlands; De Zorgboog, Postbus 16, Bakel 5760 AA, The Netherlands; Geriatric Rehabilitation Centre Topaz Revitel, Bargelaan 198, Leiden 2333 CW, The Netherlands; ParView, Interim Management and Organisational Advice, Voorsterweg 124, Brummen 6971 KC, The Netherlands; Oktober, Wielewaal 10, Bladel 5531 LJ, The Netherlands; De Zorgboog, Postbus 16, Bakel 5760 AA, The Netherlands; Department of Neurology, Erasmus MC University Medical Center, Postbus 2040, Rotterdam 3000 CA, The Netherlands; Rotterdam Stroke Service, Nieuwe Binnenweg 33, Rotterdam 3014 GB, The Netherlands

**Keywords:** geriatric rehabilitation, quality indicators

## Abstract

Quality of care is an essential aspect of geriatric rehabilitation. Usually, there are national standards for the quality of care or indicators to measure the quality of care. However, this is not the case for geriatric rehabilitation. Therefore, the aim of this study was to develop structure, process, and outcome indicators to measure the quality of geriatric rehabilitation.

To develop quality indicators for geriatric rehabilitation, a literature search was performed to identify indicators for all types of rehabilitation that can also be suitable for geriatric rehabilitation. Thereafter, in the qualitative phase, different stakeholders were inte. Indicators from the literature and indicators developed based on the interviews were merged and processed in a questionnaire. Through this questionnaire, elderly care physicians and managers of geriatric rehabilitation facilities were asked to rate the indicators on relevance and feasibility. Indicators that were considered relevant and feasible by the respondents were included in the final quality indicator set for geriatric rehabilitation.

Thirty-six indicators suitable for geriatric rehabilitation were identified from the literature. Additionally, 55 quality indicators were developed based on the interviews. Merging the indicators and omitting duplicates resulted in 69 quality indicators. Analysis of the data from the questionnaires resulted in a final set of 27 quality indicators for geriatric rehabilitation that consists of 17 structure, 8 process, and 2 outcome indicators.

This study contributes to the quality of geriatric rehabilitation by providing a first set of quality indicators ready to use in practice. Follow-up research is recommended and may include an assessment of the applicability, reliability, and validity of the developed indicator set.

## Introduction

The population aged >65 years worldwide increased from 382.5 million in 1980 to 962.3 million in 2017 and is expected to increase to 2.1 billion in 2050 [1]. An ageing population is associated with increased multimorbidity and geriatric syndromes such as impaired cognition, frailty, gait, and balance problems, leading to an increased risk of disabilities [2–4]. Additionally, patients with multimorbidity and geriatric syndromes are more likely to be hospitalized [5]. Forty per cent of frail and older people (>70 years) are hospitalized at some moments in life [5, 6]. In 2020, 51 830 (∼7% of the hospitalized elderly) patients in the Netherlands were referred to geriatric rehabilitation after hospitalization [7]. An international average number of patients referred to a geriatric rehabilitation facility after hospitalization is challenging to determine since the organizational structure of geriatric rehabilitation differs per country.

Geriatric rehabilitation is defined as a multidimensional approach of ‘diagnostic and therapeutic interventions, the purpose of which is to optimize functional capacity, promote activity and preserve functional reserve and social participation in older people with disabling impairments.’ [8] Possible diagnoses for geriatric rehabilitation are, e.g. cerebrovascular accident, heart failure, chronic obstructive pulmonary disease, amputations, or elective orthopaedics. The primary goal of geriatric rehabilitation is that rehabilitants return to their home situation; on average, 73% of geriatric rehabilitants accomplish this goal. If this is not possible, other options regarding follow-up care will be considered, such as admission to a nursing home or hospice [9]. Geriatric rehabilitation is complex, and many health-care professionals are involved in the care process since rehabilitants have different diseases, conditions, and symptoms and, therefore, different needs regarding treatment. The rehabilitation team comprises therapists and rehabilitation workers, such as occupational therapists, physical therapists, psychologists, social workers, speech and language therapists, dietitians, nurses, and physicians [10].

To assess the quality of geriatric rehabilitation, certain domains should be identified. The World Health Organization determined seven quality domains. Health care should be safe, effective, efficient, timely, people-centred, equitable, and integrated [11]. To judge whether the quality of geriatric rehabilitation is sufficient based on these domains, quality criteria and tools to measure the quality can be used. Usually, there are (inter)national standards for the quality of care (which care providers must meet) or indicators to measure the quality of care. Indicators can give organizations direction and provide information about the status of the quality. Indicators also inform organizations on which aspects the quality of care can be improved. Colsen and Casparie define an indicator as ‘a measurable aspect of care that indicates the quality of care’ [12]. The Dutch organization for elderly care physicians, Verenso, developed seven performance indicators for geriatric rehabilitation concerning the themes—effectiveness (discharge destination and rehabilitation effectiveness), patient experiences (quality of life, care control, and satisfaction), expertise (competence), and integrated care (agreement) [13]. Although indicators are relevant, there were no specific process, structure, and outcome indicators to measure the quality of geriatric rehabilitation found in the literature. It is important that these indicators are developed to improve the quality of geriatric rehabilitation [14–16]. Also, health insurers underline the importance of a set of quality indicators for geriatric rehabilitation. According to health insurers, indicators could ensure affordable, accessible, and qualitative good geriatric rehabilitation. Therefore, this study aims to describe the development of quality indicators to measure the quality of geriatric rehabilitation.

## Methods

This study aims to develop structure, process, and outcome indicators to measure the quality of geriatric rehabilitation. Typically, well-designed indicators are developed based on the literature that indicates which factors influence health-care quality. However, for some types of health care, no standards or best practices are available. In this case, indicators can be developed based on the consensus using an expert panel or consensus process [17].

### Study design

For this study, a mixed-methods design was used. First, a literature search was performed to identify existing indicators for all types of rehabilitation which are possibly applicable to geriatric rehabilitation. Second, a qualitative study design was applied to explore if additional indicators could be identified. The Consolidated criteria for Reporting Qualitative research checklist was used to ensure that important items of qualitative research were considered. The third part of this study was quantitative, whereby the sets with indicators from the literature and qualitative research were merged and proposed to managers and elderly care physicians of organizations that provide geriatric rehabilitation. The indicator set was developed using the steps of the Indicator Development Manual of the Dutch Institute for Health Care Improvement Centraal Begeleidings Orgaan (CBO) [18]. The CBO manual is based on the Appraisal of Indicators through Research and Evaluation instrument [19]. The indicator set was developed using the following steps: (i) establishing the overall goal of the indicator development, (ii) composing a research/working group, (iii) re-establishing the overall goal in the research/working group, (iv) clearly defining the scope, (v) searching for indicators in literature, (vi) identifying indicators from interviews with stakeholders, (vii) listing potential indicators, (viii) summarizing potential indicators, (ix) elaborating indicators into a questionnaire, (x) reviewing indicators and assessing relevance and feasibility, and (xi) adapting and finalizing indicators [19]. Ethical approval was not deemed necessary for this study.

### Literature search

#### Data collection

A literature search was performed using Scopus, PubMed, and Google Scholar. The literature search aimed at finding existing quality indicators that may apply to geriatric rehabilitation. Different search terms were used: geriatric rehabilitation, quality geriatric rehabilitation, indicators geriatric rehabilitation, indicators rehabilitation, quality rehabilitation, effectivity rehabilitation, effectivity rehabilitation elderly, effectivity geriatric rehabilitation, effective geriatric rehabilitation, and cost-effectiveness geriatric rehabilitation. The literature search was performed in English and Dutch, and there was no limitation concerning the publication date.

### Qualitative study

#### Study population

Nurses, managers, elderly care physicians, and health insurers involved in the care process of rehabilitants in geriatric rehabilitation facilities were interviewed to identify quality indicators. The population of nurses, managers, and elderly care physicians was selected with purposive sampling at two organizations. Also, representatives of two Dutch health insurers were interviewed. Both are regarded as large insurers with 2 and 5 million insured people, respectively, and are therefore purposefully selected. The interviewees were approached by telephone and email. The transcripts of four interviews with different health-care professionals were analysed before conducting other interviews. No new categories emerged; therefore, additional interviews were deemed unnecessary since there was a code saturation.

#### Data collection

The interviews were conducted by B.V. using video calls via applications Skype, Teams, or Zoom, dependent on the preference of the interviewee. During the interviews, B.V. had a BSc degree in Health Sciences and was finalizing the study Health Sciences and Business Administration (MSc). No relationship was established before the study’s commencement with the respondents. The respondents were informed about the research and the aim of the interview and were asked for informed consent for audio recording. During the interviews, an interview guide based on the literature review was used to ensure that predetermined topics would be discussed (Appendix 1). The interviews lasted ∼30 min, and there were no non-participants present during the interviews. The interview guide was pilot tested before the interviews were conducted. The interview guide was not provided to the participants, and the transcripts were not returned to participants for comments or corrections. At the end of each interview, a summary was given by B.V. to ensure that the information was well understood.

#### Data analysis

The interviews were processed into intelligent verbatim transcripts. The first step was open coding, which resulted in 123 different labels. The second step was axial coding, resulting in a list of 32 categories. The last step was selective coding, in which core categories were developed. The different coding phases were performed by B.V. and reviewed by V.Z. and J.G.V.M. J.G.V.M. worked at the University of Twente as an assistant professor and had experience as a senior researcher at different organizations. V.Z. has extensive experience in project management, change management, and research in health-care and geriatric rehabilitation. The three researchers reached a consensus concerning various labels. Quality indicators for geriatric rehabilitation were developed based on the labels attached to the different categories. Suitable labels were translated into structure, process, or outcome indicators. For example, the label ‘Patients should be aware of the discharge criteria at the start of the rehabilitation’ of the category ‘Discharge’ was transformed into the structure indicator ‘Discharge criteria are discussed at admission’. B.V. and V.Z. performed this phase of indicator development, and a consensus was achieved during this phase.

### Quantitative

#### Study population

All 146 health-care organizations that provide geriatric rehabilitation in the Netherlands were approached to participate in this study and were sent the questionnaire with quality indicators. A manager or elderly care physician of every organization was contacted. When the manager or elderly care physician was not able to respond to the questionnaire, another suitable professional working in geriatric rehabilitation was allowed to complete the questionnaire. Contact details of the manager and elderly care physician of the organization were provided by the commissioning party of this study, ParView.

#### Data collection

The indicators that were identified from the literature and the indicators that were developed based on the interviews were combined and merged into a questionnaire. All identified indicators were used, and duplications were removed. B.V. performed this step, and V.Z. reviewed the process. A document with background information was sent along with the questionnaire. Additionally, this document included a guideline with information about how to fill in the questionnaire and a definition of relevance and feasibility. Also, the difference between structure, process, and outcome indicators was provided. The questionnaire was conducted online using Qualtrics, and every 2 weeks, a reminder was sent to the managers and elderly care physicians who did not respond to the questionnaire. After 8 weeks, the data collection was stopped. Using a 9-point Likert scale, respondents were asked to criticize the level of agreement per indicator regarding the relevance and feasibility. An indicator is considered relevant when the indicator reflects the quality of geriatric rehabilitation, and the health-care provider can influence the outcome of the indicator. An indicator was considered feasible when the required data are available or can be made available and when the required time and effort to collect the data are acceptable.

#### Data analysis

The output of the questionnaires was imported from Qualtrics into SPSS. The answers of respondents who started the questionnaire but did not complete the entire questionnaire were included in the data analysis. An analysis (numbers, percentages, and median) per indicator was performed to define how respondents rated the indicators based on relevance and feasibility. A percentage of respondents who rated the relevance and feasibility in the highest category (7, 8, or 9) was calculated. When the median of relevance and feasibility was seven or higher, and the percentage of respondents who assessed the indicator as relevant and feasible was ≥70%, the indicator was considered appropriate and was selected for the final set of quality indicators.

## Results

### Literature search

**Figure 1:**
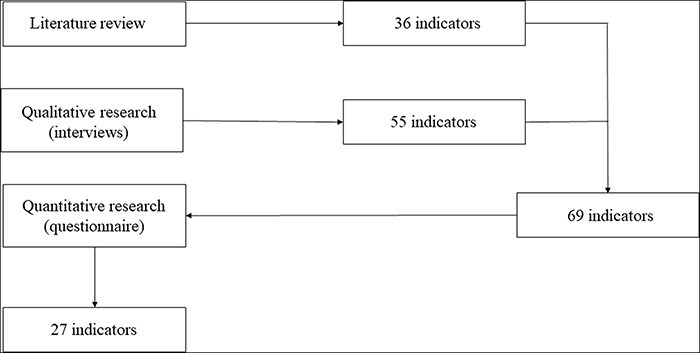
Process of indicator development.

Indicators of all types of (rehabilitation) care that can be applicable to geriatric rehabilitation were identified from five articles, which resulted in 36 indicators (Fig. 1) [20–24]. Indicators in the final indicator set derived from the literature are indicated with the letter L in Table 1.

**Table 1.** Quality indicators for geriatric rehabilitation.

**Structure indicators**
IND1: an unambiguous triage model is used (Q)
IND2: an individual multidisciplinary rehabilitation plan is designed for each rehabilitant (L) [20]
IND3: health-care providers should be aware of the fact that a patient has to do as much as possible themselves in the context of ‘everything is rehabilitation’ (Q)
IND4: rehabilitants are involved in the development of the rehabilitation plan (L, Q) [20, 21]
IND5: informal caregivers are involved in the rehabilitation process (Q)
IND6: informal caregivers are present during treatment by a physiotherapist or occupational therapist (Q)
IND7: rehabilitants receive information (digitally or on paper) about the disease and rehabilitation process (Q)
IND8: discharge criteria are discussed at admission (Q)
IND9: valid medical devices are used (L) [20]
IND10: medical devices are inspected annually (Q)
IND11: medication is prescribed through an electronic prescription system (L) [21]
IND12: a culture in which all incidents are reported prevails (Q)
IND13: staff has to be adequately qualified to provide geriatric rehabilitation (L, Q) [21, 24]
IND14: an elderly care physician specialized in rehabilitation medicine is employed (Q)
IND15: an elderly care physician is 24/7 on call (Q)
IND16: at least one health-care psychologist must be active within the geriatric rehabilitation (Q)
IND17: there must be 24-h availability of registered nurses (Q)
**Process indicators**
IND18: average length of stay per diagnosis group (L, Q) [22]
Numerator: total length of stay
Denominator: total number of rehabilitants
IND19: treatment intensity per diagnosis group (L, Q) [23]
Numerator: total hours of treatment per diagnosis group
Denominator: total number of rehabilitants per diagnosis group
IND20: % admission interviews where informal caregivers of the rehabilitant were present (Q)
Numerator: number of admission interviews where informal caregivers of the rehabilitant were present
Denominator: total number of admission interviews
IND21: % of rehabilitants whose medication was verified upon admission (L) [21]
Numerator: number of rehabilitants whose medication was verified at admission
Denominator: total number of admitted rehabilitants
IND22: % of rehabilitants screened for malnutrition at admission (L) [21]
Numerator: number of rehabilitants screened for malnutrition at admission
Denominator: total number of admitted rehabilitants
IND23: % of rehabilitants whose medication was verified at discharge (L) [21]
Numerator: number of rehabilitants whose medication was verified at discharge
Denominator: total number of discharged rehabilitants
IND24: % incident reports that have been systematically analysed (L, Q) [20]
Numerator: number of incident reports that have been systematically analysed
Denominator: total number of incident reports
IND25: % of practitioners that annually participates in training, education, or courses (Q)
Numerator: number of practitioners who annually participates in training, education, or courses
Denominator: total number of practitioners
**Outcome indicators**
IND26: % of rehabilitants returning home per diagnosis group (Q)
Numerator: number of rehabilitants returning home
Denominator: total number of admitted rehabilitants (calculated per diagnosis group)
IND27: % of rehabilitants satisfied with the care received (L, Q) [21]
Numerator: number of rehabilitants who completed Netto Promoter Score (NPS) positive (≥6)
Denominator: total number of rehabilitants who completed the rehabilitant satisfaction survey

IND: indicator; L: derived from literature; Q: derived from qualitative research.

### Qualitative results

For the qualitative part of this research, two nurses, two managers, and two elderly care physicians from two organizations that provide geriatric rehabilitation were interviewed. Also, two experts, each from two different health insurers, were interviewed. The first open coding phase of the transcripts resulted in 123 different labels that contained information concerning the quality of geriatric rehabilitation. During the second coding phase, 30 categories were created based on the labels. During the last coding phase, 30 different categories were attached to seven core categories. Based on the labels suitable for developing quality indicators, 55 different quality indicators for geriatric rehabilitation were developed (Fig. 1). Indicators in the final indicator set derived from the interviews are indicated with the letter Q in Table 1.

### Quantitative results

The indicators from the literature and those developed based on the interviews were merged, which resulted in a set of 69 indicators (Fig. 1). These 69 indicators were processed in a questionnaire. The questionnaire with quality indicators was sent to all 146 health-care organizations that provide geriatric rehabilitation in the Netherlands. Sixty-six respondents (45%) started with the questionnaire, and 44 completed the questionnaire. The occupations of the respondents are given in Table 2. The respondents worked in 50 different organizations; hence, 34% of the organizations that provide geriatric rehabilitation in the Netherlands participated in this study. Based on the questionnaire, 27 quality indicators for geriatric rehabilitation were selected for the final indicator set. Table 1 presents the final indicator set with quality indicators for geriatric rehabilitation.

**Table 2.** Occupation of the respondents of the quantitative research.

Occupation	*n* (%) (*N* = 66)
Manager	24 (36.4)
Elderly care physician	15 (22.7)
Elderly care physician specialized in rehabilitation	15 (22.7)
Policy officer	1 (1.5)
Case manager	1 (1.5)
Nurse	5 (7.6)
Director	1 (1.5)
Speech and language therapist	1 (1.5)
Process manager	2 (3.0%)
Physical therapist	1 (1.5)

### Final results

The indicators from the literature and the indicators that were developed based on the interviews in this study, were merged. This resulted in a set of 69 indicators (Figure 1). These 69 indicators were processed in a questionnaire. The questionnaire with quality indicators was sent to 146 health care organizations in geriatric rehabilitation. Based on the questionnaire, 27 quality indicators for geriatric rehabilitation were selected for the final indicator set (Table 1).

## Discussion

### Statement of principal findings

A set of quality indicators for geriatric rehabilitation was developed on a national level. The indicators were developed using a mixed-methods approach, combining literature, qualitative, and quantitative research. There are four important reasons to measure the quality of geriatric rehabilitation using indicators [14–16]. First, providers of geriatric rehabilitation have the desire to use quality indicators to improve the quality of health care. Second, there is a need for quality indicators to justify to health insurers which (quality of) health care has been provided [16]. Third, the quality indicators can be used as a benchmark, and as a result, best practices can be established [25, 26]. Fourth, patients can benefit from these quality indicators because they can select their preferred provider and benefit from a higher quality of care [25].

In the quantitative part of this study, the questionnaire was sent to managers, elderly care physicians, and elderly care physicians who specialized in rehabilitation. In the questionnaire, the occupation of respondents was asked. An equal number of respondents from each group responded. Two types of experts were approached since it was important to include different stakeholders with different opinions. All stakeholders may have different opinions and interests regarding rehabilitation. For example, an elderly care physician focuses more on the patient, while a manager focuses on the effectiveness of care and damage burden. Nevertheless, on the occupational level, the groups did not answer the questionnaire differently. This indicates that the experts overall have the same opinions concerning the relevance and feasibility of quality indicators for geriatric rehabilitation, regardless of occupation.

The response rate of the questionnaire was 45%. Many doctors and managers responded to the invitation mail that they had no time to answer the questionnaire since they were too busy with the coronavirus disease of 2019 pandemic. Considering this pandemic, the response rate of 45% is regarded as sufficient. On average, the response rate from individuals in studies with questionnaires is 53% [27]. The response rate in this study on the organizational level is 34%. The average response rate from organizations in studies with questionnaires is 36% [27]. Therefore, the response rate in this study is considered sufficient and similar to the average response rate of studies with questionnaires [27]. Representativity can be an issue, given the response rate. It is advisable to do follow-up research at a later stage to obtain a higher response rate.

The final indicator set contains 27 indicators. The indicators are divided into structure, process, and outcome indicators. The researchers have carefully classified the indicators into one of these domains. However, the domains can be multi-interpretable. This should be taken into account when using the indicators.

### Strengths and limitations

First, developing indicators with different stakeholders is a strength of this study since it resulted in a set of quality indicators that reflects the opinion of different health-care professionals with different points of view. Second, 34% of the organizations that provide geriatric rehabilitation in the Netherlands participated in this study. Third, feasibility and relevance were considered. Feasibility is essential because data availability is required to calculate the indicators, and the indicators must be applicable in practice. Also, relevance is important since quality indicators must measure aspects that represent the quality of geriatric rehabilitation. Relevance also represents the ability to influence the outcome of the indicators [12, 17]. When the median of relevance and feasibility was seven or higher, and the percentage of respondents that assessed the indicator as relevant and feasible was ≥70%, the indicator was considered appropriate and was selected for the final set of quality indicators. After careful consideration, these cut-off points of 7 and 70% were set by the researchers to reach a number of indicators similar to other indicator sets in health care. The set of quality indicators for medical specialist rehabilitation includes 25 indicators, the indicator set for long-term nursing home care includes 27 different indicators, and for diabetes, there are 26 quality indicators [28–30]. A set of quality indicators must be concise. Lower cut-off points would result in a large indicator set that is not workable in daily practice. However, higher cut-off points would result in an indicator set in which too few indicators are included, possibly missing important quality aspects. A few indicators that the authors regarded as valuable failed on the feasibility criterion. The authors carefully considered this aspect but wanted the indicator set manageable. Additionally, the authors wanted to avoid influencing the results by intuitively adding indicators to the final indicator set.

Paramedics were not included in the qualitative part of this study; this was a limitation of the study. However, two paramedics responded to the questionnaire in the quantitative part of this study. Herewith, the opinion of paramedics is partly included in the indicator set. The paramedics did not have a divergent opinion compared to other respondents in the quantitative part. The second limitation is that this study did not include rehabilitants and their informal caregivers.

### Interpretation within the context of the wider literature

The indicator set developed in this study can be used particularly to measure the quality of geriatric rehabilitation in detail. Compared to the five articles from the literature search, the final list of indicators includes indicators specifically for geriatric rehabilitation. The final list contains 15 (out of 27) indicators not reported in the five articles. Various databases have been consulted, but no other quality indicator sets for geriatric rehabilitation were found in the literature. However, in 2013, a workgroup of elderly care physicians and a patient organization formulated a set of seven performance indicators for geriatric rehabilitation [13]. Comparing the Verenso performance indicator set with the quality indicator set developed in this study, two indicators are not included in the set with quality indicators in this study: (i) performance indicator ‘efficiency’ with operationalization ‘functional improvement by Barthel index’ and (ii) performance indicator ‘patient experiences’ with operationalization ‘quality of life’. These indicators were originally available; however, from the participant’s point of view, these indicators failed on the criterion for feasibility used in this study. Patient experiences are included in the indicator that was developed in this study with the NPS indicator. The NPS is a metric in which patient satisfaction can be expressed. A possible explanation for the absence of a quality of life indicator is that the quality of life may be less important for short-term rehabilitation and is more suitable for ambulatory and chronic care. Also, the Barthel index is not included in the indicator set. A possible reason is that the indicator set developed in this study focuses on the quality of geriatric rehabilitation, and the Barthel index is more associated with the effectiveness of geriatric rehabilitation.

### Implications for practice, policy, and research

Organizations that provide geriatric rehabilitation can use this indicator set to monitor, benchmark, and improve the quality of care. Affiliates in the Geriatric Rehabilitation E-cademy expressed the willingness to implement this set of indicators in their organizations.

### Conclusions and recommendations

This study contributes to the quality of geriatric rehabilitation by providing a first set of quality indicators ready to use in practice. Further research can focus on whether this set of quality indicators is valid and reliable. In order to assess this set of quality indicators on applicability, reliability, and validity, data are required. Therefore, the set of quality indicators developed in this study has to be used in practice to collect data.

## Supplementary Material

mzad044_Supp

## Data Availability

The data underlying this article cannot be shared publicly due to the privacy of individuals who participated in the study. The data will be shared on reasonable request to the corresponding author.

## Appendix 1 Interview schemes

**Geriatric doctor/nurse**: what are your tasks regarding the care for geriatric patients who rehabilitate in the field of……

‘Topic 1, Therapeutic treatment, patient care, and patient education’

- How can it be shown that a treatment (medical procedure) is of good quality?
- How can it be shown that the care (normal, daily care) for the patient is of good quality?
- Which aspects are important for the education of patients?

‘Topic 2, Medical-technical equipment’

- Which aspects are important when using medical equipment on patients?

‘Topic 3, Internal quality’

- Do situations arise that you would have handled differently afterwards?
- Do colleagues share experiences and points of improvement (systematically) with each other?

‘Topic 4, Staffing’

- How does the staff contribute to a good quality of care?

‘Topic 5, General’

If (one of) the following six characteristics have (has) not yet been discussed during the interview, discuss the relevant characteristic(s).

Good quality of care meets six characteristics, how can it be ensured that geriatric rehabilitation:

- Is safe? (harming the patient is prevented)
- Is effective? (care is provided according to scientifically proven treatments and misuse of care is prevented)
- Is efficient? (waste of care is prevented)
- Is patient-centred? (care is organized around the patient, with respect for the patient’s wishes)
- Is equitable? (all patients have equal right, no one should be favoured)

Did you miss anything during this interview? Has something not been discussed that is important for the quality of geriatric rehabilitation?

**Manager: how do you guarantee the quality of the following aspects?**

**Health insurer: what is important for the quality of the following aspects?**

‘Topic 1, Therapeutic treatment, patient care, and patient education’

- How can it be shown that a treatment (medical procedures) is of good quality?
- How can it be shown that the care (normal, daily care) for the patient is of good quality?
- Which aspects are important for the education of patients?

‘Topic 2, Medical-technical equipment’

- Which aspects are important when using medical equipment on patients?

‘Topic 3, Internal quality’

- How can lessons be learned from mistakes made, and how can these mistakes be prevented in the future?

‘Topic 4, Staffing’

- How does the staff contribute to a good quality of care?

‘Topic 5, General’

If (one of) the following six characteristics have (has) not yet been discussed during the interview, discuss the relevant characteristic(s)

Good quality of care meets six characteristics, how can it be ensured that geriatric rehabilitation:

- Is safe? (harming the patient is prevented)
- Is effective? (care is provided according to scientifically proven treatments and misuse of care is prevented)
- Is efficient? (waste of care is prevented)
- Is patient-centred? (care is organized around the patient, with respect for the patient’s wishes)
- Is equitable? (all patients have equal right, no one should be favoured)

Did you miss anything during this interview? Has something not been discussed that is important for the quality of geriatric rehabilitation?
