# Supplementary material for: The development of indicators to measure the quality of care in geriatric rehabilitation
Source: Int J Qual Health Care. 2023 Sep 1;36(1):mzad044. doi: 10.1093/intqhc/mzad044 (PMC10914440; doi:10.1093/intqhc/mzad044)
Supplement: mzad044_Supp [file mzad044_supp.zip › Appendix 1.docx]

# Appendix 1 Interview schemes

**Geriatric doctor/nurse**: What are your tasks regarding the care for geriatric patients that rehabilitate in the field of……

*Topic 1, Therapeutic treatment, patient care, and patient education*

- How can it be shown that a treatment (medical procedure) is of good quality?
- How can it be shown that the care (normal, daily care) for the patient is of good quality?
- Which aspects are important for the education of patients?

*Topic 2, Medical-technical equipment*

- Which aspects are important when using medical equipment on patients?

*Topic 3, Internal quality*

- Do situations arise that you would have handled differently afterwards?
- Do colleagues share experiences and points of improvement (systematically) with each other?

*Topic 4, Staffing*

- How does the staff contribute to a good quality of care?

*Topic 5, General*

If (one of) the following six characteristics has not yet been discussed during the interview, discuss the relevant characteristic(s)

Good quality of care meets six characteristics, how can it be ensured that geriatric rehabilitation:

- Is safe? (harming the patient is prevented)
- Is effective? (care is provided according to scientifically proven treatments and misuse of care is prevented)
- Is efficient? (waste of care is prevented)
- Is patient-centred? (care is organized around the patient, with respect for the patient’s wishes)
- Is equitable? (all patients have equal right, no one should be favoured)

Did you miss anything during this interview? Has something not been discussed that is important for the quality of geriatric rehabilitation?

**Manager: how do you guarantee the quality of the following aspects:**

**Health insurer: what is important for the quality of the following aspects:**

*Topic 1, Therapeutic treatment, patient care, and patient education*

- How can it be shown that a treatment (medical procedures) is of good quality?
- How can it be shown that the care (normal, daily care) for the patient is of good quality?
- Which aspects are important for the education of patients?

*Topic 2, Medical-technical equipment*

- Which aspects are important when using medical equipment on patients?

*Topic 3, Internal quality*

- How can lessons be learned from mistakes made, and how can these mistakes be prevented in the future?

*Topic 4, Staffing*

- How does the staff contribute to a good quality of care?

*Topic 5, General*

If (one of) the following six characteristics has not yet been discussed during the interview, discuss the relevant characteristic(s)

Good quality of care meets six characteristics, how can it be ensured that geriatric rehabilitation:

- Is safe? (harming the patient is prevented)
- Is effective? (care is provided according to scientifically proven treatments and misuse of care is prevented)
- Is efficient? (waste of care is prevented)
- Is patient-centred? (care is organized around the patient, with respect for the patient’s wishes)
- Is equitable? (all patients have equal right, no one should be favoured)

Did you miss anything during this interview? Has something not been discussed that is important for the quality of geriatric rehabilitation?
