# Supplementary material for: The development of indicators to measure the quality of care in geriatric rehabilitation
Source: Int J Qual Health Care. 2023 Sep 1;36(1):mzad044. doi: 10.1093/intqhc/mzad044 (PMC10914440; doi:10.1093/intqhc/mzad044)
Supplement: mzad044_Supp [file mzad044_supp.zip › suppl_data/Supplementary material not for review.docx]

**Supplementary material**

| **COREQ criteria** | **Description** |
| --- | --- |
| Interviewer | Bram Veneberg |
| Credentials | BSc, at the time that the interviews were conducted |
| Occupation | Student, at the time that the interviews were conducted |
| Gender | Male |
| Experience and training | BSc Health Sciences, various courses in interview techniques, at the time that the interviews were conducted |
| Relationship established | There was no relationship established prior to study commencement |
| Participant knowledge of the interviewer | The participants knew that the interviewer was conducting this research for his master thesis about geriatric rehabilitation. Participants also knew that the interviewer had a BSc in Health Sciences and was studying at the University of Twente. |
| Interviewer characteristics | Reasons and interests in the research topics were reported |
| Methodological orientation and Theory | Literature review/content analysis |
| Sampling | Purposive |
| Method of approach | Email |
| Sample size | 8 |
| Non-participation | None |
| Setting of data collection | Remote using e.g. MS teams, Skype |
| Presence of non-participants | There were no non-participants present |
| Description of sample | Doctors, nurses, managers, health insurers |
| Interview guide | An pilot tested interview guide was used (Appendix 1) |
| Repeat interviews | No repeat interviews were carried out |
| Audio/visual recording | Audio recording was used to collect the data |
| Field notes | Field notes were made during the interviews |
| Duration | Approximate 60 minutes |
| Data saturation | Yes, code saturation |
| Transcripts returned | Transcripts were not returned to participants |
| Number of data coders | Two |
| Description of the coding tree | A short description of the coding tree is provided |
| Derivation of themes | Themes were identified in advance and derived from the data |
| Software | Microsoft Office Word |
| Participant checking | Participants had the opportunity to provide feedback on the results in the quantitative part of this study |
| Quotations presented | Quotations were not presented in this article |
| Data and findings consistent | There is consistency between the data presented and the findings |
| Clarity of major themes | Major themes are clearly presented in the findings |
| Clarity of minor themes | Minor themes were not included in the quantitative part of this study and therefore not discussed since they seem less relevant according to the aim of this study |
